# Genomic Characterisation of *Limosilactobacillus fermentum* CRL2085 Unveiling Probiotic Traits for Application in Cattle Feed

**DOI:** 10.1111/1758-2229.70176

**Published:** 2025-09-08

**Authors:** Cecilia M. Aristimuño Ficoseco, Daniele Chieffi, Marco Montemurro, Annarita Bavaro, Carlo G. Rizzello, Maria E. F. Nader‐Macias, Silvina Fadda, Francesca Fanelli, Vincenzina Fusco, Graciela M. Vignolo

**Affiliations:** ^1^ Reference Center for Lactobacilli (CERELA‐CONICET) San Miguel de Tucumán Argentina; ^2^ National Research Council, Institute of Sciences of Food Production (CNR‐ISPA) Bari Italy; ^3^ Department of Environmental Biology “Sapienza” University of Rome Rome Italy

**Keywords:** exopolysaccharide, folate, genomics, *Limosilactobacillus fermentum*, probiotic, riboflavin

## Abstract

*Limosilactobacillus fermentum* CRL2085, isolated from feedlot cattle rations, displayed high efficiency as a probiotic when administered to animals. A comprehensive genomic analysis was performed to elucidate the genetic basis underlying its probiotic potential. Fifteen genomic islands and CRISPR‐Cas elements were identified in its genome. Pan‐genomic analysis highlighted the dynamic evolution of this species, and clustering based on the nucleotide genomic similarity only partially correlated with the source of isolation or the geographic origin of the strains. Several genes known to confer probiotic properties were identified, including those related to adhesion, resistance to acidic pH and bile salts, tolerance to oxidative stress, metabolism/transport of sugars and other compounds, and genes for exopolysaccharide biosynthesis. In silico analysis of antimicrobial resistance genes and virulence determinants confirmed the safety of this strain. Moreover, genes related to B‐group vitamins biosynthesis and feruloyl esterase hydrolase were also found, showing the nutritional contribution of the strain, which also showed moderate adhesion capability, exopolysaccharide production when grown with sucrose, and the capacity to metabolise 42 out of 95 carbon substrates tested. This data provides the genetic basis for deciphering the mechanisms beyond the benefits demonstrated by its use during cattle intensive raising and confirms its promising role as a probiotic.

## Graphical abstract

A comprehensive genomic and phenotypic analysis of *Limosilactobacillus fermentum* CRL2085 revealed key probiotic and nutritional traits supporting its safe application in cattle feed. The strain harbors genes for stress tolerance, adhesion, exopolysaccharide production, and B‐vitamin biosynthesis, and efficiently metabolizes plant‐derived carbohydrates, indicating its potential to enhance nutrient availability and gut health in livestock.

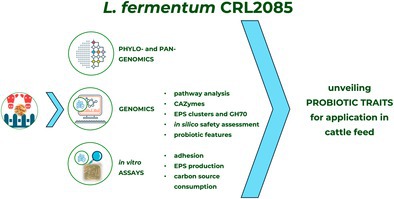

## 1 Introduction

*Lactobacillus* genus is a group of Gram‐positive, non‐sporulating, facultative anaerobic bacteria that are normally found in nature and can be isolated from different matrices, such as fermented foods, plant materials, dairy‐related products, human and animal intestine (Ayivi et al. 2020; Heeney et al. 2018; Maldonado et al. 2018; Mota‐Gutierrez and Cocolin 2021; Rodriguez‐Sanchez et al. 2021). Description of this genus was amended by Zheng et al. (2020), and currently the *Lactobacillaceae* family includes 36 child taxa. *Limosilactobacillus* (*L*.) *fermentum* comb. nov., previously known as *Lactobacillus fermentum* , is the type species of the *Limosilactobacillus* gen. nov. described in 2020 (Zheng et al. 2020) and included strains previously belonging to the *Lactobacillus cellobiosus* species, from which it was differentiated in 2004 (Dellagio et al. 2004). However, *L. fermentum* is the only species in the *Limosilactobacillus reuteri* group rarely found in intestinal ecosystems but frequently isolated from plants and spontaneously fermented cereals (Gänzle and Ripari 2016), being an example of a species undergoing reversion of the lifestyle from host adapted to free living species (Duar et al. 2017). It is a heterofermentative microorganism and has a widespread ecology, being naturally found in the human/animal gastrointestinal tract, human vagina and dental caries, dairy products, silage, and plant fermented food and beverages. It is included in the list of microorganisms generally recognised as safe (GRAS) and with Qualified Presumption of Safety (QPS) by the Food and Drug Administration (FDA) and European Food Safety Authority (EFSA), respectively. It produces a hydrolase enzyme (BSH) which makes it resistant to bile salts through the elimination of the conjugated amino acid fraction of bile acids (Dong and Lee 2018). *L. fermentum* strains are used as starter culture due to their relevant technological properties, such as exopolysaccharides production and antimicrobial activity (Pakroo et al. 2022). Starter cultures of this species have improved the nutritional value, flavour and functional properties of fermented milk, plants and silage (Okoye et al. 2023). Moreover, *L. fermentum* has been recognised as a probiotic species and applied in humans and animals due to its anti‐inflammatory and immunomodulatory properties (Rodríguez‐Sojo et al. 2021).

The Food and Agriculture Organisation and the World Health Organisation (FAO/WHO) define probiotics as “live microorganisms that, when ingested in adequate amounts, confer a health benefit on the host” (FAO/WHO 2002; Hill et al. 2014; ISAPP 2018). The microorganisms most used as probiotics are lactic acid bacteria (LAB), which are generally considered harmless microorganisms and are used as starter cultures to ferment food matrices for domestic and commercial purposes (Ayivi et al. 2020). In addition, LAB are found in the gastrointestinal system of humans and animals, which is physiologically important for intestinal health (Pasolli et al. 2020). While probiotic strains include the genera *Bifidobacterium*, *Pediococcus*, *Lactococcus*, and *Enterococcus*, most probiotics currently on the market belong to the genus formerly known as *Lactobacillus*, a taxonomically heterogeneous group reclassified into 25 genera including more than 170 species (Zheng et al. 2020).

In intensive cattle raising and/or fattening systems (feedlots), the main objective is to obtain quality meat accompanied by high animal productivity through efficient growth performance (Bhogoju and Nahashon 2022). A common practice to promote the growth of animals for meat production is the use of antibiotics as growth promoters in animal feed, which, over long periods, has contributed to the global crisis of antimicrobial resistance by the emergence of antibiotic‐resistant bacteria and difficulty in treating infections in animals/humans. In 2006 and 2017, the EFSA and the FDA (EC 2003; U.S. FDA 2013), respectively, banned the use of antibiotics as animal growth promoters. In the search for alternatives, the use of probiotics/prebiotics/symbiotic and phytobiotics, among others, emerged as alternatives for antimicrobials (Al‐Shawi et al. 2020; Bąkowski and Kiczorowska 2021). The EFSA has published guidelines for the safety assessment of probiotics, such as taxonomic identification of the strain through whole genome sequencing, genomic and phenotypic determination of antibiotic resistance profile and virulence factors (Koutsoumanis et al. 2021). The preliminary evaluation of the probiotic potential of a newly isolated strain is thus crucial before proceeding to the assessment of its health benefits and use within a formulation in food and pharmaceutical products (Da Silva et al. 2024). *L. fermentum* strains isolated from fruit and vegetables have recently been positioned as novel candidates for probiotic use to promote host health benefits and biotherapeutics development (de Luna Freire et al. 2024).

*L. fermentum* CRL2085 was isolated from feedlot cattle rations, selected for its beneficial characteristics and further applied as a probiotic in feedlot cattle, where its administration has been demonstrated to improve the nutritional status, the overall productive performance and reduction of *E. coli* O157:H7 shedding (Aristimuño Ficoseco et al. 2018; Maldonado et al. 2018; Mansilla et al. 2020, 2022, 2023). The objective of this study was to perform a comprehensive genomic and phenotypic characterisation of *L. fermentum* CRL2085 in order to elucidate the genetic basis of its probiotic potential. In particular, we investigated the ability of this strain to adhere to Caco‐2 cells, produce exopolysaccharides, ferment numerous carbon sources, and screen its genome for the presence of genes involved in the metabolism and transport of sugars and other compounds. Finally, we investigated the presence of virulence and antibiotic resistance genes, allowing novel insights to support the safe and consistent utilisation of this strain as probiotic in cattle feed.

## 2 Materials and Methods

### 2.1 Strain and Culture Conditions

*L. fermentum* CRL2085 was isolated from feedlot cattle rations and selected for its beneficial characteristics (Aristimuño Ficoseco et al. 2018; Maldonado et al. 2018). It was stored at −80°C in milk–yeast extract (13% non‐fat milk, 0.5% yeast extract, and 1% glucose) and glycerol 20%. The inoculum of the strain was prepared by transferring stock cultures to MRS broth (Merck, Darmstadt, Germany) and sub‐cultured twice in the same medium at 37°C for 16 h.

### 2.2 Whole Genome Sequencing (WGS)

Total DNA was obtained according to Pospiech and Neumann (1995). The integrity, purity and quantity of DNA were assessed by agarose gel electrophoresis, Nanodrop photometer (Peqlab) and Qubit 3.0 fluorometer (by using Qubit dsDNA Quantification Assay Kits, Life Technologies). Whole genome shotgun sequencing was performed by Novogene Bioinformatics Technology Co. Ltd. (Beijing, China) on a NovaSeq platform (Illumina, San Diego, USA) by using the Illumina mate pair with the 2x250 mate pair procedure (Nextera Mate Pair Library Preparation Kit, Immumina, San Diego, USA), according to the manufacturer's instructions. Reads were then trimmed by the NxTrim (V2) (O'Connell et al. 2015) and de novo assembly was performed using the genome assembler SKESA (Souvorov et al. 2018) implemented in the Read assembly and Annotation Pipeline Tool (RAPT; https://github.com/ncbi/rapt). The whole genome shotgun projects have been deposited at DDBI/ENA/GenBank under the accession JAXKWT000000000. The version described in this paper is JAXKWT010000000.

### 2.3 Bioinformatic Methods

#### 2.3.1 WGS Analysis

The overall contiguity of the assembly and genome statistics was determined with the Genome Taxonomy Data Base (GTDB) Anchor pipeline implemented in The Microbial Genomes Atlas (MIGA; https://disc‐genomics.uibk.ac.at/miga; Rodriguez‐R et al. 2018). The completeness of the de novo assembly was measured evaluating the presence of 111 essential single‐copy genes that are observed across almost all prokaryotic genomes (Rodriguez‐R et al. 2018), while contamination was measured by the frequency at which these genes are present in more than one copy. The quality score was then calculated as completeness percentage minus five times contamination percentage.

#### 2.3.2 Gene Prediction, Phylogenomic and Comparative Analysis

*L. fermentum* CRL2085 genes were predicted and annotated by using the NCBI Prokaryotic Genome Annotation Pipeline (PGAP; Tatusova et al. 2016) implemented in the Read assembly and Annotation Pipeline Tool (RAPT; https://www.ncbi.nlm.nih.gov/rapt/), the Comprehensive Genome Analysis Service implemented into the Bacterial and Viral Bioinformatics Resource Centre (BV‐BRC; https://www.bv‐brc.org/) platform, and by the PROKKA pipeline (Seemann 2014), and then manually curated; protein ID used in the manuscript indicated those obtained by using PGAP. Phylogenetic analysis was performed employing the Bacterial Genome Tree service implemented in the BV‐BRC (version 3.35.5; https://www.bv‐brc.org/) with the Maximum Likelihood method RAxML (version 8.2.11) and progressive refinement (Stamatakis 2018). For the analysis, we used 40 genomes representative of all 33 validly published species included in the *Limosilactobacillus* genus (Table S1). Phylogeny was inferred on 192 single‐copy genes identified in the genomes of *Limosilactobacillus* spp. used in this analysis. *Paucilactobacillus oligofermentans* DSM 15707 was used as an outgroup. Visualisation of the phylogenetic trees was performed by using iTOL (version 6.9; Letunic and Bork 2024). Comparative genomic analysis of 96 *L. fermentum* strains (Table S1) was performed using the PanACoTA (PANgenome with Annotations, COre identification, Tree and corresponding Alignments; Perrin and Rocha 2021) software implemented in Pan‐genome Explorer (Dereeper et al. 2022; https://panexplorer.southgreen.fr/) with minimum percent identity for BLAST of 80%.

### 2.4 In Silico Evaluation of Probiotic Potential

The Basic Local Alignment Search Tool (BLAST) algorithms were used to perform homology‐based analysis toward reference sequences (http://blast.ncbi.nlm.nih.gov/Blast.cgi). The evaluation of probiotic potential of *L. fermentum* CRL2085 was performed by homology‐based analysis with reference proteins associated with probiotic features in the *Limosilactobacillus* genus (Guhanraj and Dhanasekaran 2024; Phujumpa et al. 2022; Ullah et al. 2024). Homology was predicted by BLASTP with a cutoff E value of 1e^10^ and minimum identity of 35% and then manually curated. Active carbohydrate enzymes were identified by executing CAZyme annotation on the dbCAN3 meta server (Zheng et al. 2023; http://bcb.unl.edu/dbCAN2/blast.php). Putative gene clusters involved in the biosynthesis of ribosomally synthesised and post‐translationally modified Peptides (RiPPs) and (unmodified) bacteriocins were predicted with antiSMASH (version 7.0; Blin et al. 2023), while Phastest (PHAge Search Tool with Enhanced Sequence Translation; https://phastest.ca/; Wishart et al. 2023) web server was used to identify prophage sequences within the bacterial genome. Genomic Islands were predicted by using the IslandViewer webserver (Bertelli et al. 2017; https://www.pathogenomics.sfu.ca/islandviewer/). Contigs were rearranged using *L. fermentum* strain SCB0035 as the reference genome. The CRISPRCasFinder webserver (https://crisprcas.i2bc.paris‐saclay.fr/; Couvin et al. 2018) was used to detect CRISPR direct repeats and spacers. The Resistance Gene Identifier tool implemented in The Comprehensive Antibiotic Resistance Database (CARD; https://card.mcmaster.ca/home) was used to predict *L. fermentum* CRL2085 resistome. In addition, K‐mer based detection method for antimicrobial resistance genes was performed by using the Comprehensive Genome Analysis Service of BV‐BRC. Proteins associated with antimicrobial resistance were also retrieved by keyword search within the UniProt ID entry list obtained by functional annotation. Unless specified, tools were used with default parameters.

### 2.5 EPS Production

*L. fermentum* CRL2085, *Leuconostoc pseudomesenteroides* 20193^T^ (positive control), and *Weissella diestrammenae* DSM 27940^T^, previously characterised as a no‐EPS producing strain (negative control; Fanelli et al. 2023), were grown on agar plates containing modified de Man, Rogosa, and Sharpe medium (MRS, Oxoid, Italy) supplemented with sterile sugar (purity > 99.5%, Sigma, Merck, Germany) solutions to obtain a final concentration of 20 g/L of either glucose, sucrose, fructose, or galactose. All strains were cultivated for 24 h as previously described and inoculated by streaking in triplicate on MRS‐sugar agar media. After incubation at 37°C for 48 and 72 h, the strains that produced slimy colonies were recorded as capable of producing EPS.

### 2.6 Carbon‐Sources Utilisation

The carbon‐source utilisation pattern by *L. fermentum* CRL2085 was determined using Biolog AN microplates system (Biolog Inc., Hayward, CA, USA), containing 95 different carbon sources. Briefly, the strain was grown in MRS broth (Oxoid, Italy) for 24 h. Cells were then collected by centrifugation (10,000 rpm, 10 min), washed two times with sterile phosphate buffer (50 mmol/L pH 7.0) and then resuspended in sterile physiological saline solution (0.9 w/v NaCl). Each plate well was inoculated with 100 μL of the bacterial suspension adjusted to 65% transmittance and subsequently incubated at 37°C for 24 h anaerobically as recommended by the manufacturer. Positive reactions were automatically recorded using the MicroStation microplate reader (Biolog) at 590 nm and 750 nm wavelength.

### 2.7 Adhesion Assay

The adhesion assay was performed as described by Fanelli et al. (2023), with slight modifications. Caco‐2 cells were cultured in Dulbecco's Modified Essential Medium High Glucose (DMEM; Euroclone S.p.A, Italy) supplemented with 10% inactivated fetal bovine serum, 1% L‐glutamine, 1% antibiotic‐antimycotic solution (Euroclone S.P.A, Italy), and 1% non‐essential amino acids solution (Sigma‐Aldrich, Italy) at 37°C in a humidified atmosphere containing 5% CO_2_. Cells were harvested twice a week up to 70%–80% confluence using a trypsin–EDTA solution. Cell density and viability were determined using LUNA‐II Automated Cell Counter (Logos Biosystem) and the cells used in the experiments showed a mean viability of 90%. Caco‐2 cells were seeded in 12‐well cell culture plates (3.85 cm^2^) at a density of 5 × 10^4^ cells/cm^2^ (1.9 × 10^5^ cells/mL) and incubated at 37°C with 5% CO_2_ for 7 days. The cell culture medium (1 mL/well) was refreshed every 3 days to achieve complete confluence of cells. *Lacticaseibacillus rhamnosus* GG (ATCC 53103) was used as a positive control due to its known binding capacity to intestinal cells. For the adhesion assay, bacterial cultures grown overnight in MRS broth at 37°C were refreshed in MRS broth and incubated at 37°C. After 4 h, the cultures were adjusted to an optical density corresponding to ca. 10^8^ CFU/mL as previously determined. Serial dilutions of the suspension were plated on MRS agar to calculate the initial viable bacterial counts (CFU/mL) (I). One mililiter of this suspension was collected by centrifugation (5000 rpm, 10 min), washed with Dulbecco's phosphate buffered saline (PBS) without calcium and magnesium (Euroclone S.p.A, Italy), and resuspended in the DMEM without supplements. Caco‐2 cell monolayers were then washed twice with Dulbecco's PBS without calcium and magnesium (Euroclone S.p.A, Italy), and 1 mL of the bacterial suspension was added to each well. After incubation for 2 h at 37°C with 5% CO_2_, unattached bacteria were removed by washing the monolayers three times with PBS. To lyse the cells, the monolayers were treated for 15 min with 1 mL of 1% (v/v) Triton X‐100, and serial dilutions of the resultant lysates were plated on MRS agar to calculate the number of adhered bacteria (A). The % of adhesion was calculated according to the formula: % adhesion = [A (CFU/mL)/I (CFU/mL)] × 100.

### 2.8 Statistics

For the adhesion assay, the results of the experiments carried out in triplicate were expressed as mean % of adhesion ± standard error. The *t* test (*p* < 0.05) was performed to evaluate statistically significant differences.

## 3 Results

### 3.1 General Features of *L. fermentum* CRL2085 Genome

**FIGURE 1:**
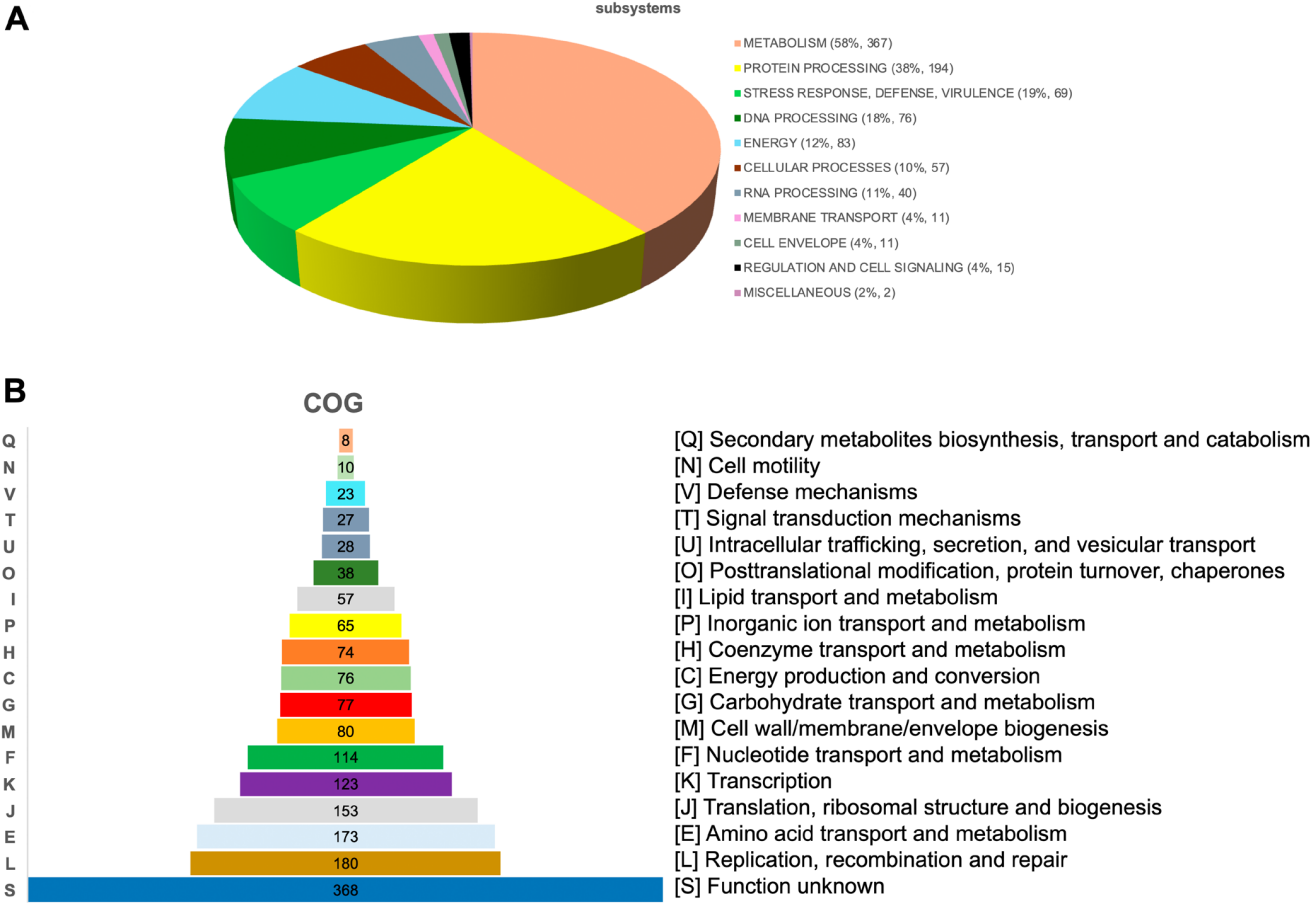
(A). Subsystems and associated genes counted in the *L. fermentum* CRL2085 genome. (B) COGs functional classification of genes annotated in *L. fermentum* CRL2085.

General features of the *L. fermentum* CRL2085 genome are listed in Table 1. The assembled genome has a total length of 1,914,364 bp, a N50 of 49.5 kb, an average GC content of 52.22%, and 1941 coding genes with a coding density of 85.91%. The quality of the assembly was excellent and no plasmid sequences in the genome were identified. The annotated genome included 476 hypothetical proteins and 1465 proteins with functional assignments as shown in Figure 1A. The highest number of genes was counted in the subsystem metabolism (367), followed by protein processing (194), energy (83), DNA processing (76), and stress response, defence, virulence (69) and cellular processing (57). In addition, the EggNOG mapper classification assigned the highest number of genes from *L. fermentum* CRL2085 to the category replication, recombination, and repair (180), followed by amino acid transport and metabolism (173), translation, ribosomal structure, and biogenesis (153), transcription (123), and nucleotide transport and metabolism (114) (Figure 1B). A total of 15 genomic islands were predicted in the genome of *L. fermentum* CRL2085 (Table S2). They include genes involved in carbohydrate metabolism, such as glycosyltransferases and polysaccharide biosynthetic proteins (island 2), the riboflavin biosynthetic genes (island 5), a cluster of CRISPR‐associated proteins (islands 6 and 9), and the putative cluster of terpenes (squalene/phytoene) polyisoprene synthase proteins biosynthesis (island 13). Nevertheless, various integrases/recombinases and 81 transposases were also detected in the CRL2085 genome (Table S3). No complete intact prophagic region was predicted by Phastest, while seven sequences with CRISPR and 148 Cas elements were identified in the genome of *L. fermentum* CRL2085 (Table S4).

**TABLE 1:** Genomic feature of *L. fermentum* CRL 2085.

Total length	1,914,364 bp
Number of scaffolds	129
Largest contig	119,510 bp
N50	36,114
GC content	52.22
Predicted genes	1957
CDS	1890
tRNA	54
ncRNAs	3
rRNAs	2, 6, 2 (5S, 16S, 23S)
Pseudo genes	72
Coding density %	85.91
Quality	Excellent

### 3.2 Phylogenomic and Comparative Genomic Analysis

**FIGURE 2:**
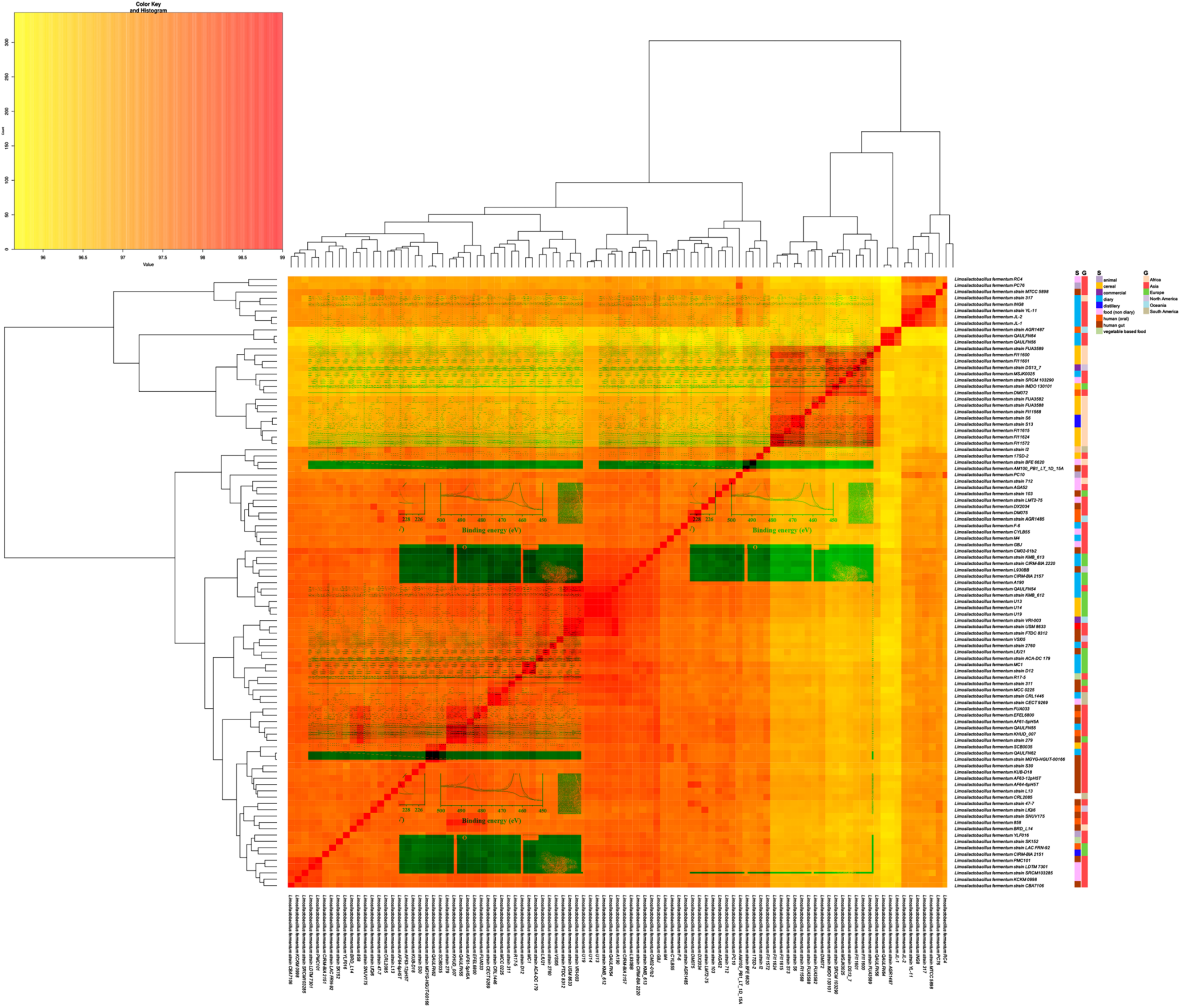
Whole‐genome sequence comparisons and heatmap of the Average Nucleotide Identity among *L. fermentum* strains. Isolation source and geographical origin are indicated by a square box close to the strain name, according to the legend.

Phylogenetic analysis confirmed the taxonomic placement of the strain CRL2085 and the localization of this species in the genus obtained by Zheng et al. (2020), close to *L. gorilla* species (Figure S1). Comparative genomic analysis was performed on 96 genomes of *L. fermentum* (Table S1) by Pan‐Explorer that identified a pangenome size of 7948 clusters, with 771 core genes, 4576 dispensable genes, and 2601 strains specific genes. The genetic divergence, based on the average nucleotide identity (ANI) among the analysed strains is shown in Figure 2.

On average, the strains shared 98.24% of nucleotide identity, and *L. fermentum* AF615pH5A resulted in the closest genome with respect to *L. fermentum* CRL2085 (Table S5), with a value of 98.97% of ANI. As shown in Figure 2, the clustering inferred appears to be only partially correlated with the geographical origin or the source of isolation of the strains. Clustering based on Euclidean distance using the accessory genomes of *L. fermentum* strains is shown in Figure S2. The comparison was based on the presence/absence of softcore, dispensable, and singleton gene clusters; overall, the distribution of the 2004 gene clusters showed that they grouped independently from their geographical origin or isolation source; *L. fermentum* CRL2085 is located close to strains PMC101, isolated from human vagina, and BFE6620, isolated from fermented cassava, and near to strains isolated from food and human faeces, or cereal‐associated strains.

### 3.3 In Silico Evaluation of Probiotic Potential

#### 3.3.1 Carbohydrate‐Active Enzymes (CAZymes)

As reported in Table 2, the genome of *L. fermentum* CRL2085 harbours several genes coding for putative CAZymes. The 34 CAZymes predicted by dbCAN3 (HMMER prediction) mainly belonged to the glycosyltransferases (GTs) family with 18 genes among them; 7 genes from the GT2 family, 5 from GT4, and 2 from GT51 were counted, followed by the glycoside hydrolases (GHs) family, which showed 15 genes including 5 from the GH73 family, while 1 gene was assigned to the Auxiliary Activity Family 1 (AA). The most prevalent enzymes identified were GT2 (7 genes) involved in the biosynthesis of disaccharides, oligosaccharides, and polysaccharides, followed by GT4 (5 genes) which involved many glycoside transferases that utilise diverse nucleotide‐sugar donors in the glycosidic bond formation.

**TABLE 2:** CAZYmes identified in *L. fermentum* CRL2085 genome.

Class	Product	Protein ID
AA1	Multicopper oxidase domain‐containing protein	UIR13_00650
GH13_18	Sucrose phosphorylase	UIR13_07390
GH13_29	Alpha, alpha‐phosphotrehalase	UIR13_07725
GH13_31	Alpha‐galactosidase	UIR13_02585
GH179	Gfo/Idh/moca family oxidoreductase	UIR13_00625
GH2	Glycoside hydrolase family 2 TIM barrel‐domain containing protein	UIR13_03460
GH32	Sucrose‐6‐phosphate hydrolase	UIR13_09125
GH36	Alpha‐galactosidase	UIR13_01325
GH65	Glycoside hydrolase family 65 protein	UIR13_05660
GH70	Glycoside hydrolase family 70 protein	UIR13_07035
GH73	Lysm peptidoglycan‐binding domain‐containing protein	UIR13_01170
	Glucosaminidase domain‐containing protein	UIR13_02830
	Glycoside hydrolase family 73 protein	UIR13_00510
		UIR13_08875
		UIR13_09225
GH8	Glycosyl hydrolase family 8	UIR13_08365
GT111	DUF4422 domain‐containing protein	UIR13_00810
GT113	Galactofuranosyltransferase	UIR13_02790
GT14	Beta‐1,6‐N‐acetylglucosaminyltransferase	UIR13_05075
GT2	Glycosyltransferase family 2 protein	UIR13_00830
		UIR13_02785
		UIR13_02800
		UIR13_02810
		UIR13_05040
		UIR13_05060
	Glycosyltransferase	UIR13_08360
GT28	Undecaprenyldiphospho‐muramoylpentapeptide beta‐N‐acetylglucosaminyltransferase	UIR13_09365
GT4	Accessory Sec system glycosyltransferase Asp1	UIR13_03860
	Glycosyltransferase	UIR13_03865
		UIR13_05035
	Glycosyltransferase family 4 protein	UIR13_07090
		UIR13_07095
GT51	Transglycosylase domain‐containing protein	UIR13_07295
	PBP1A family penicillin‐binding protein	UIR13_08800

#### 3.3.2 Genes Associated With Probiotic and Nutritional Function

Genes associated with putative probiotic functions are listed in Table S6. Among them, genes coding for stress response elements (11) such as the molecular chaperon and co‐chaperon *groEL* and *groES* genes; adhesion (3), biofilm formation (11), and aggregation (2) such as genes coding for the *inlJ* with a MucPB domain, *mapA* coding gene for a collagen binding protein, and a *luxS* gene that participates in the quorum‐sensing system with impact on biofilm development; acid tolerance (10) involving several proton pump coding genes; oxidative stress survival (3) which encompasses cold shock protein and peroxidases coding genes; exopolysaccharides biosynthesis (9) including *galE* and *galU* genes coding for the UDP‐glucose 4‐epimerase GalE and the UTP‐glucose‐1‐phosphate uridylyl‐transferase GalU respectively, a beta‐phosphoglucomutase, and phosphoglucosamine mutase, among others. In relation to the nutritional function, six genes implicated in folate biosynthesis (*fol*A, *fol*B, *fol*C, *fol*E, *fol*K, and *fol*P), genes (6) for the complete pathway for riboflavin biosynthesis (ribAB, ribD, ribE, ribF, ribH, and yigB), and the genes (2) coding for feruloyl esterase activity which improve bioavailability of iron, mineral, and dietary fibre content were detected; while genes coding for alcohol tolerance (16), immunomodulation (5), supporting function (1), GI survival/stress response (3), bile salt tolerance (31), extreme temperature tolerance (2), osmotic shock tolerance (4), cell wall formation (6), moonlighting proteins (6) and glucose metabolism (1) were additionally harboured by the *L. fermentum* CRL2085 genome.

#### 3.3.3 Prediction of Secondary Metabolites Production

Gene involved in secondary metabolites production were predicted by using antiSMASH, which identified a putative cluster of 20.5 kb nt (contig 14:35,263–55,709) for terpene biosynthesis (island 13; Table S2), which included several transposases and recombinases/integrases. The core biosynthetic gene, coding for a squalene/phytoene synthase family protein, was, however, predicted as a pseudogene (UIR13_01345).

#### 3.3.4 In Silico Safety Assessment

Genes associated with potential antimicrobial resistance phenotype are listed in Table 3. These included the genes coding for two tetracycline resistance genes (*tetA*, *tetO*), three penicillin‐binding proteins genes (*pbp2b*, *ponA, pbpB*), a penicillin V acylase (*yxeI*), two genes for multidrug transporters (*emrB, mepA*) and a bacitracin resistance protein (*uppP*). In addition, in the *L. fermentum* CRL2085 genome, UIR13_07610 protein was annotated as a VanZ family protein.

**TABLE 3:** Antimicrobial resistance associated elements predicted in *L. fermentum* CRL2085 genome.

Resistance	Gene	Protein names	Protein ID
Bacitracin	*uppP*	Bacitracin resistance protein	UIR13_08030
Beta‐lactam	*yxeI*	Penicillin V acylase	UIR13_05645
			UIR13_06225
	*pbp2b*	Penicillin‐binding protein 2B	UIR13_03015
	*ponA*	Penicillin‐binding protein 1A	UIR13_07295
	*pbpB*	Penicillin‐binding protein 2B	UIR13_09380
	*cpoA*	Alpha‐galactosylglucosyldiacylglycerol synthase	UIR13_07090
Cationic antimicrobial peptides (CAMPs)	*mprF*	LPG synthase	UIR13_07860
Fomidomycin	*fsr*	Fosmidomycin resistance protein	UIR13_00685
Macrolides	*macB*	Macrolide export ATP‐binding/permease protein MacB	UIR13_04275
Multidrug	*emrB*	Multidrug export protein EmrB	UIR13_09775
	*mepA*	Multidrug export protein MepA	UIR13_00955
			UIR13_02955
Tetracycline	*tetA*	Tetracycline resistance protein	UIR13_03655
	*tetO*	Tetracycline resistance protein TetO	UIR13_07270

### 3.4 In Vitro Probiotic Features Analysis

#### 3.4.1 EPS Production

**FIGURE 3:**
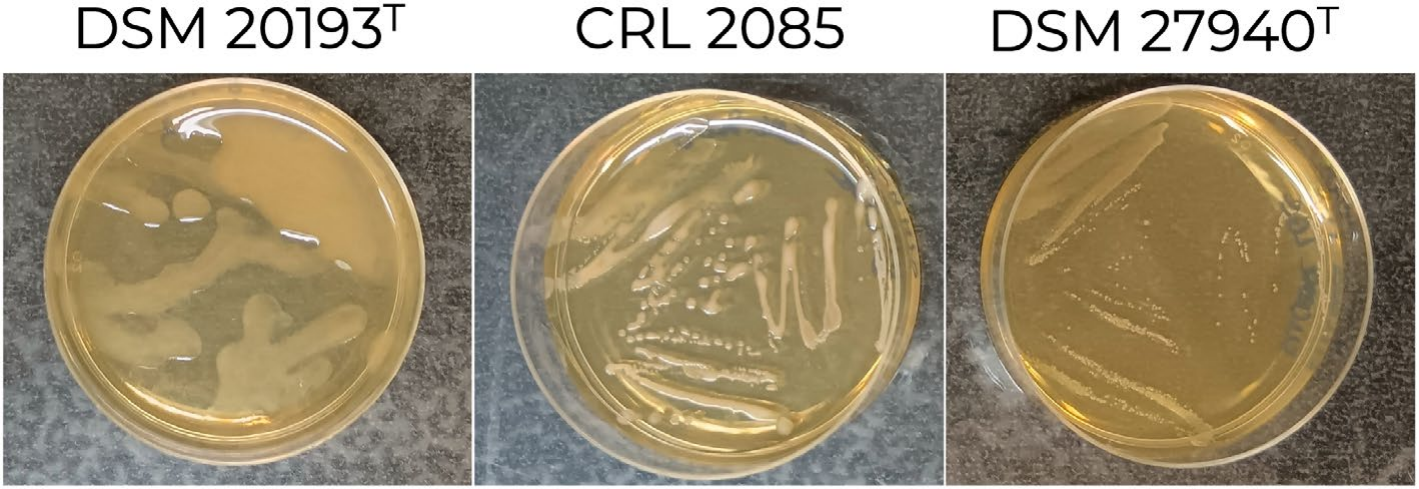
EPS production. Plates of *L. fermentum* CRL2085, *Leuconostoc pseudomesenteroides* DSM 20193^T^, used as a positive control, and *Weissella diestrammenae* DSM 27940^T^, used as a negative control, grown in MRS supplemented with 20% sucrose at 48 h.

As shown in Figure 3, *L. fermentum* CRL2085 was able to produce clear ropy and viscous material in MRS supplemented with sucrose medium after 48 h of incubation. No EPS production was visible in MRS supplemented with glucose, fructose, or galactose (data not shown).

#### 3.4.2 Carbon‐Source Utilisation Pattern

**FIGURE 4:**
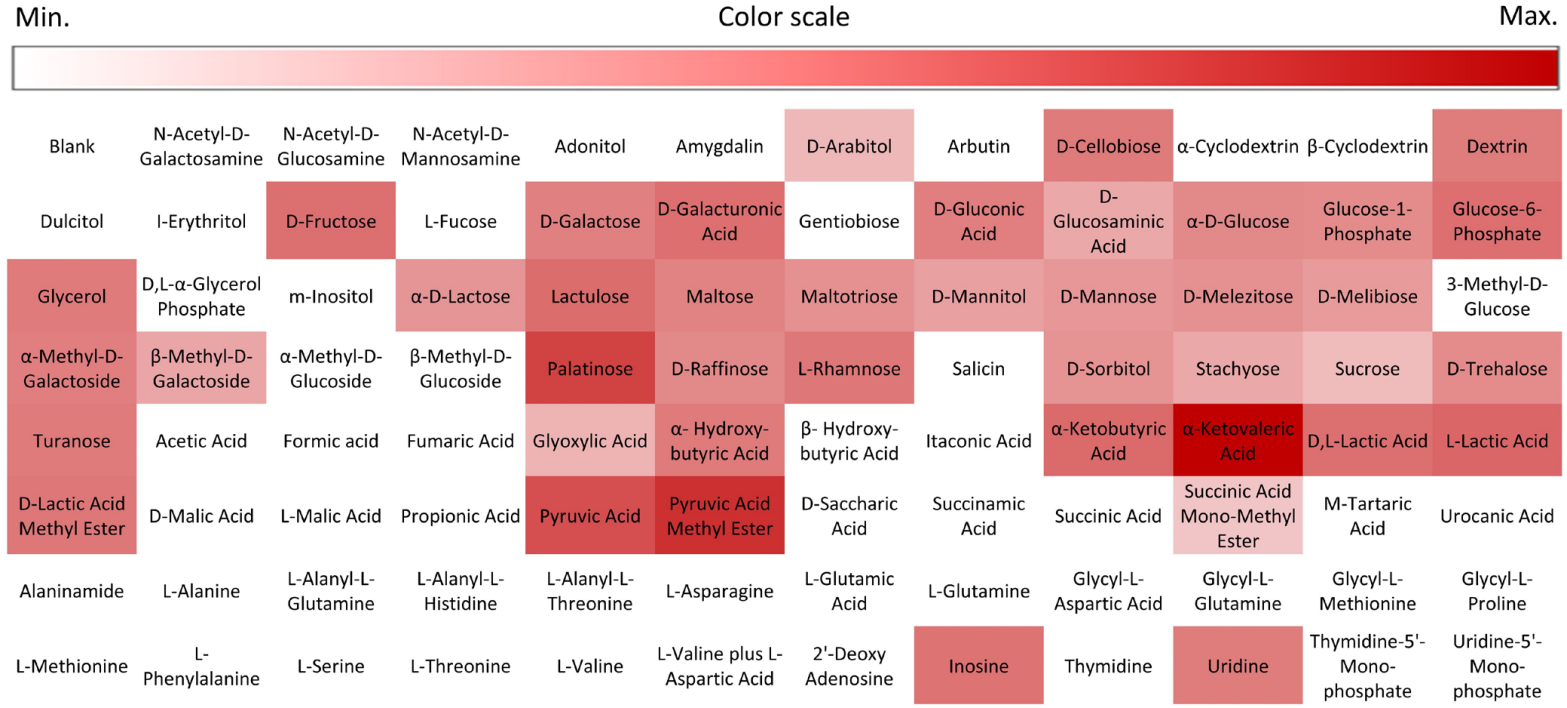
Carbon source utilisation or oxidation by *L. fermentum* CRL2085 using AN MicroPlate (Biolog Inc., Hayward, CA, USA). The colour scale indicates the metabolism of each carbon source ranging from white (no utilisation or oxidation) to dark red (maximum utilisation or oxidation).

To characterise the carbon substrate utilisation patterns of *L. fermentum* CRL2085, the AN Biolog micro plates were used (Figure 4). Even with great differences, 42 out of 95 hydrocarbon substrates were consumed by the probiotic strain. The utilisation pattern of carbon sources was greatest for α‐ketovaleric acid, palatinose, and pyruvic acid methyl ester; also d‐cellobiose, d‐fructose, d‐galacturonic acid, glucose‐6‐phosphate, lactulose, l‐lactic acid, and pyruvic acid were highly consumed by the CRL2085 strain. Utilisation was also observed for the remaining 32 substrates, where positive consumption was detected, while in the other 53 substrates no reaction was revealed.

#### 3.4.3 Adhesion Assay

*L. fermentum* CRL2085 was tested for its ability to adhere to the Caco‐2 cell monolayer. It exhibited a moderate‐low adhesion of 0.32% ± 0.22% (*p* < 0.05) compared to the probiotic *L. rhamnosus* GG, which was used as a positive control and showed an adhesion of 2.54% ± 0.55%.

## 4 Discussion

*Limosilactobacillus fermentum* CRL2085 isolated from feedlot cattle rations has been identified and characterised as tolerant to GIT conditions, with adhesive and biofilm‐forming ability, producer of antimicrobial compounds (acid and H_2_O_2_) and feruloyl‐esterase enzyme related to nutrient degradation (Maldonado et al. 2018; Uezen et al. 2020). In addition, although phenotypic sensitivity to all assayed antibiotics was exhibited by this strain, the occurrence of ERY resistance genes was previously reported (Aristimuño Ficoseco et al. 2018). The use of this probiotic strain in feedlot cattle contributed to improving metabolic‐nutritional status, overall productive performance, and reduction of *E. coli* O157:H7 shedding (Mansilla et al. 2022, 2023, 2024). Thus, as a promising candidate for antibiotic replacement in feedlot cattle, *L. fermentum* CRL2085 genome sequencing analysis and in vitro experiments were performed to elucidate the genetic basis underlying its probiotic potential.

The genome has a total length of 1.91 Mb, with an average GC content of 52.22% and excellent assembly quality. No plasmid sequences were identified in the genome. The GC content and genome size of CRL2085 were similar to other strains, including *L. fermentum* DSM20052 from fermented beets (GC 52.50%; 1.89 Mb; Brandt et al. 2020), IMD0130101 strain from rye sourdough (GC 51.50%; 2.09 Mp; Verce et al. 2018), SNUV175 strain from vaginal tract (GC 51.50%; 2.18 Mb; Lee et al. 2017), MTCC25067 strain from dahi fermented milk (GC 51.46%; 1.95 Mb; Aryantini et al. 2017), among others. The GC content of this species is generally higher than the typical low GC of the strains belonging to genera that were formerly included in the genus *Lactobacillus* (Brandt and Barrangou 2018), suggesting that *L. fermentum* might have experienced less genomic changes. Loss of ancestral genes and metabolic simplification are central trends in the evolution of LAB. Major gene loss has occurred in the common ancestors of *Lactobacillales*, indicating early adaptation to nutritionally rich environments; however, genome decay seems to be an ongoing process, as all species show the loss of specific genes (Makarova et al. 2006).

In this study, *L. fermentum* core genome was reported to be relatively small, representing 9.7% of the total, and the number of pan genes increased by adding further genomes to the analysis, without reaching a plateau (data not shown). This indicates that this species has an open pangenome, which is characteristic of species for which the genomic heritage is still not completely defined, and whose diversity increases with constant acquisition of genes, creating a genetic repertoire which evolves and provides opportunities for adaptation to wider environmental niches and hosts (Ksiezarek et al. 2022; Zhao, Yu, et al. 2022). Here, the proximity of strains derived from different geographic and isolation sources inferred by the comparative analysis is consistent with similar investigations recently performed by Zhao, Yu, et al. (2022) and might indicate that some *L. fermentum* strains have been recently introduced in certain environments. Moreover, the genomic plasticity of CRL2085 was demonstrated by the presence of 15 genomic islands, several integrases/recombinases and 81 transposases (Tables S2 and S3) which, especially in food‐derived strains, indicate major exposure to mobile genetic elements (Zhao, Yu, et al. 2022) and the acquisition of novel genes to improve its gene pool. In addition, no hit for virulence determinants and no complete intact prophagic regions were predicted. Prophage remnants lack the number of genes necessary to be classified as fully functional phage. This agrees with the findings reported by Pei et al. (2021), who found prophage fragments in almost all 16 *Lactobacillus* (*sensu lato*) species, with intact prophages detected at lower frequencies. Although an uneven distribution of prophages among the analysed species was noted, multihabitat species retained more prophages in their genomes than habitat‐restricted species. Also, seven sequences with CRISPR were identified in the genome of *L. fermentum* CRL2085. CRISPR sequences are short, highly conserved repetitive regions in the genome that are interspersed with spacers (variable sequences) and are often located adjacent to CRISPR‐associated (Cas) genes (Hille and Charpentier 2016). The presence of these CRISPR‐Cas systems can contribute to strain genetic stability, as they can prevent the natural transformation with foreign nucleic acid fragments, as well as infection and bacteriophage conjugation (Samson et al. 2015). Compared with other bacteria, LAB harbours plenty of CRISPR‐Cas systems, which are present in approximately 63% of lactobacilli (Crawley et al. 2018). This system contributes to generating safer and more robust strains with increased resistance against bacteriophage and prevents the dissemination of plasmids carrying antibiotic‐resistance markers. Furthermore, the CRISPR‐Cas system from LAB could be used to exploit novel, programmable genome editing tools of native host and other organisms, resolving the limitation of genetic operation of some LAB species, increasing the important biological functions of probiotics, improving their adaptation in complex environments, and inhibiting the growth of foodborne pathogens, as was recently reported (Cui and Qu 2024).

The phylogenomic and pangenomic analysis performed revealed high diversity among *L. fermentum* strains. The obtained clustering only partially reflected the isolation source or geographical origin of the strains. Indeed, although *L. fermentum* CRL2085 was isolated from feedlot rations (containing cereals such as corn grains and corn silage and sorghum), the other members of the clade were of human/animal, dairy, and plant origin. The same evidence emerged from the presence/absence of clusters in the accessory genomes, where *L. fermentum* strains showed a lack of partitioning depending on their isolation source indicative of niche specialisation, apart from a just outlined grouping with a prevalence of strains from cereal‐associated environments (Figure S2). The promiscuity of *L. fermentum* strains isolated from different sources may suggest that some strains moved into the human microbiome through food sources as a transient member rather than a permanent member. As such, it may explain the lack of specific niche adaptation of *L. fermentum* (Duar et al. 2017). Our results agree with previous studies (Verce et al. 2020; Phujumpa et al. 2022), by which this species was hypothesised to undergo a reversion from its host‐adapted lifestyle as a species of *L. reuteri* group to a free‐living nomadic lifestyle. This is evidenced by its isolation from highly diverse environments, such as plant material fermentations, fermented milk, human vagina/saliva, and human/animal faeces, but also manure and sewage (Kim et al. 2023; Lee et al. 2017; Pakroo et al. 2022; Phujumpa et al. 2022; Zhao, Zhang, et al. 2022).

Our genomic analysis indicated the presence of the *emrB* gene coding for a multidrug export protein, but no additional streptomycin resistance gene was annotated (Table 3). In addition, two genes encoding tetracycline resistance (*tetA* and *tetO*), the *mepA* gene encoding a multidrug export protein, the *macB* gene encoding a noncanonical ABC transporter with transmembrane domains (TMD) forming a pore in the inner membrane, and an ATP‐binding domain (NBD) responsible for energy generation were predicted. The membrane protein MacB is an ABC‐type macrolide efflux transport system that confers resistance against macrolides composed of 14‐ and 15‐membered lactones when overexpressed (Kobayashi et al. 2001). Despite the genetic determinants potentially associated with antibiotic resistance, in a previous study, the sensitivity of *L. fermentum* CRL2085 against eight antibiotics (AMP, CLI, CHL, ERY, GEN, KAN, TET, and STR) was evaluated using the MIC value method, and the safety of this strain was confirmed (Aristimuño Ficoseco et al. 2018).

Carbohydrate metabolism was reported as a trait supporting the probiotic potential of LAB (Pugh et al. 2022). It is the main source of metabolic energy, playing a crucial role in the survival and adaptation of lactobacilli in the ecological environment by participating in cellular processes such as energy generation and stress response (Gänzle and Follador 2012). When the repertoire of enzymes able to use the wide range of substrates from cattle rations was evaluated, CAZyme annotation showed that the genome of *L. fermentum* CRL2085 harbours mostly genes from the glycosyltransferase (GT) family, followed by the glycoside hydrolase (GH) family. The annotated proteins are involved in the sugar utilisation of hexose, pentose and/or complex carbohydrates such as fructose, mannose, ribose, xylitol, xylulose, trehalose, amylose, starch, and maltodextrin. These elements are essential for bacterial adaptation to the gastrointestinal (GI) environment and interaction with the host, as they are involved in the metabolism and assimilation of complex carbohydrates that are not digested, as recently reported for breast milk oligosaccharides and gluco‐oligosaccharides (Mollova et al. 2023; Zúñiga et al. 2021; Zeng et al. 2024). GHs, together with GTs, form the main catalytic machinery for the synthesis and cleavage of glycosidic bonds. Complex carbohydrates are the main biomass constituent, and their metabolism is carried out by the large GTs family of enzymes. These enzymes catalyse the transfer of a sugar molecule from an activated donor (sugars linked to dolichol‐(pyro) phosphate or mostly nucleotide sugars) to acceptors ranging from proteins and fatty acids to other carbohydrate molecules (Taujale et al. 2020). The presence of GTs enzymes will be beneficial for the survival, competitiveness, and persistence of *L. fermentum* CRL2085 within the host. In addition, mucin‐like structures facilitating adhesion to host cell mucoproteins were reported to be produced by O‐linked glycosylation on serine catalysed by GTs (Han and Vaishnava 2023). GHs also catalyse the hydrolysis of glycosidic bonds in complex sugars. Their functions include the degradation of biomass such as cellulose, hemicellulose and starch, and they participate in antibacterial defence strategies, in pathogenesis mechanisms and in normal cellular function. Since *L. fermentum* CRL2085 has been selected to be used as a probiotic for feedlot cattle, it may be active at degrading starch (from corn/sorghum) and cellulose material (from silage) involved in the feed ration. The repertoire of *L. fermentum* CRL2085 CAZYmes counted 34 enzymes, of which GH73 (5), GT2 (7), GT4 (5), and GT51 (2) were the majority. These values are comparable to those reported by Zhao, Yu, et al. (2022), who analysed the presence of active enzyme families in 224 *L. fermentum* strains. Among glycoside hydrolases, five genes from the GH73 family that specifically cleave glycosidic bonds were predicted in the CRL2085 probiotic strain in accordance with that reported for *L. fermentum* strains from food and human gut (Zhao, Yu, et al. 2022). Among this GH family, three GH73 are able to degrade oligo‐and polysaccharides, while glucosaminidases and Lysin Motif peptidoglycan hydrolase genes related to bacterial cell division were additionally found, as previously reported for *L. fermentum* and other LAB (Hu et al. 2010; Inagaki et al. 2009). In addition, three proteins classified within the sub‐families GH13–18, GH13–29 and GH13–31 were predicted in the *L. fermentum* CRL2085 genome. The three predicted proteins, annotated as sucrose phosphorylase (UIR13_07390), alpha, alpha‐phosphotrehalase (UIR13_07725), and alpha‐galactosidase (UIR13_02585), harboured an α‐amylase domain (PFAM: PF00128), which catalyses the hydrolysis of α‐1,4‐glucosidic bonds in starch and related α‐glucans. Alfa‐amylase specificity is currently present in 42 subfamilies, including representatives of α‐amylases, α‐glucosidases, α‐1,4‐glucan branching enzymes, pullulanases, cyclodextrin glucanotransferases, 4‐α‐glucanotransferases, and oligo‐α‐1,6‐glucosidases amino acid transporters, among others (Janeček et al. 2014; Plaza‐Vinuesa et al. 2019). The presence of different α‐amylases active on starch‐related carbohydrates is consistent with a potential role of *L. fermentum* CRL2085 as a fermentative agent on starch, abundantly present in cattle rations. Extracellular α‐amylase activity was reported for various lactobacilli involving *L. fermentum* , playing an important role in the GIT of animals (Tallapragada et al. 2018). In addition, the presence of one gene encoding a protein from the GH70 family, able to synthesise α‐glucan polymers from starch and sucrose (Gangoiti et al. 2018), and α‐galactosidase from the GH13–31 subfamily and GH36 family, able to hydrolyse α‐1,6 galactoside linkages cleaving glucose subunits from starch‐derived isomaltose and maltodextrins in the *L. fermentum* CRL2085 genome, was previously reported in other *L. fermentum* strains (Verce et al. 2020; Zhao, Yu, et al. 2022).

Moreover, the GTs identified in the CRL2085 genome were distributed among seven families (GT2, GT4, GT14, GT28, GT51, GT111, GT113), GT2 and GT4 being dominant with six and five predicted genes, respectively. As shown by Zhao, Yu, et al. (2022), the GT2 family, which is involved in the biosynthesis of disaccharides, oligosaccharides and polysaccharides, was dominant in *L. fermentum* strains from food sources. These results agree with the dominance of GT2 and GT4 found in *L. fermentum* LAB‐1 and *Lactiplantibacillus plantarum* strain isolated from dairy products (Hossein 2022; Liang et al. 2023). These two GT families comprise a huge number of proteins characterised from various sources which have diverse functions, being polyspecific. The GT2 and GT4 families include many glycoside transferases which utilise diverse nucleotide‐sugar donors in glycosidic bond formation (Breton et al. 2006). As reported by Gänzle and Follador (2012), lactobacilli have the strain‐ or species‐specific ability to metabolise the disaccharides cellobiose [Glu‐β‐(1→4)‐d‐Glu], trehalose [Glu‐α‐(1→1)‐d‐α‐Glu], and the α‐d‐glucosyl‐d‐fructose isomers turanose and palatinose, this being confirmed by the carbon sources utilisation patterns in the AN Biolog assay reported for *L. fermentum* CRL2085. Cellobiose, consisting of two glucose molecules linked by a β‐(1→4) bond, is the degradation product of cellulose or related plant β‐glucans. In CRL2085 we identified the *chcB* gene (UIR13_02370), coding for a cellobiose PTS system EIIC component, which simultaneously transports cellobiose from the extracellular space into the cytoplasm and phosphorylates it to cellobiose‐6P, while the 6‐phospho‐beta‐glucosidase, which converts cellobiose‐6P into d‐glucose, was predicted as a pseudogene by PGAP (UIR13_00595) but not by PROKKA.

Other carbon sources used by *L. fermentum* CRL2085 under the consumption assay using the AN Biolog system were dextrin, maltose, maltotriose, glucose‐6‐phosphate (Gluc 6‐P), glucose‐1‐phosphate (Gluc1‐P), and d‐galactose. Several genes were identified as responsible for galactose metabolism (*gal*A, *gal*K, *gal*U, *gal*T, *gal*E, *gal*M, *pgm*, *mal*L, *lac*Z, *sac*A, *gat*C, *glk*, and *glf*). In addition, maltose phosphorilase (MalP) (UIR13_05660 from GH65 family predicted in the genome of CRL2085) is involved in the reversible phosphorolysis of maltose to D‐glucose and β‐Glc1‐P (Gao et al. 2019). This metabolic reaction does not expend ATP for Gluc 6‐P generation, being energetically more favourable than hydrolysis. During maltose metabolism of heterofermenters such as *L. fermentum*, glucose is transiently accumulated in the medium, showing that Gluc 6‐P is preferentially metabolised (Gänzle and Follador 2012). MalP is highly specific for maltose and cannot convert isomaltose, maltotriose, and maltodextrins (Nakai et al. 2009). However, the utilisation of maltodextrins is a common characteristic of lactobacilli, even though extracellular amylases are not prevalent in this group. In species adapted to starch‐rich environments, such as plant‐derived lactobacilli, pullulanases and amylopullulanases, in addition to amylases, are particularly efficient in maltose/maltodextrin utilisation (Zúñiga et al. 2021). Because the use of dextrin and genes from three GH13 subfamilies were predicted in the CRL2085 genome, the presence of oligo‐1,6‐glucosidase‐ and α‐glucosidase‐encoding genes (GH13 family), which can cleave glucose subunits from isomaltose and maltodextrins, as reported for the sourdough strain *L. fermentum* IMDO130101, was confirmed (UIR13_02585, Table 2). As stated by the AN Biolog system, the utilisation of sucrose, fructose, and lactose was also observed and agreed with the carbohydrate metabolism‐related genes annotated in the genome of *L. fermentum* CRL2085, as well as in *L. fermentum* KUB‐D18 (from chicken intestines) and *L. fermentum* CECT5716 (from human breast milk) (Phujumpa et al. 2022). The gene *sacA* coding for a β‐fructofuranosidase (UIR13_09125) which hydrolyses sucrose into glucose and fructose, while *lac*M/*lac*LM genes coding for β‐galactosidase (UIR13_03460) of the GH2 family, which catalyses the hydrolysis of lactose (Gänzle 2014; Gänzle and Follador 2012). The utilisation of d‐fructose reported by the Biolog assay is consistent with the presence of the *scr*K gene coding for a fructokinase (UIR13_00960) and two glucose‐6‐phosphate isomerases (UIR13_00965 and UIR13_02090) converting fructose 6P into Glucose 6P.

In the context of cattle feed applications, the ability of *L. fermentum* CRL2085, demonstrated by phenotypic assay and by its genomic repertoire, to degrade and utilise plant‐derived polysaccharides, such as starch, cellobiose, and maltodextrins, which are abundant in silage and feedlot rations, highlights its functional compatibility with the bovine gastrointestinal environment, contributing to the degradation of complex plant materials, to a more stable and efficient ruminal fermentation, potentially enhancing fibre digestibility and nutrient absorption in cattle, improving the performance of ruminants.

Moreover, the utilisation of glycerol by *L. fermentum* CRL2085 assessed by the AN Biolog agrees with the presence of the *glpK* gene, coding for a glycerol kinase (UIR13_02395) able to transform the triol into glycerol‐3‐P, in coincidence with that reported for *L. fermentum* strains by Verce et al. (2020).

In relation to the D‐mannose consumption, we identified *man*X, *man*Y, and *man*Z coding for the mannose PTS system EIIAB (UIR13_08705), EIIC (UIR13_087010), and EIID (UIR13_08715) components, respectively, and the *man*A gene, coding for the mannose‐6‐phosphate isomerase (UIR13_08685), converting mannose 6‐P to fructose 6‐P. Trehalose consumption is realised through the action of a trehalose‐6‐phosphate hydrolase (UIR13_07725) coded by the *tre*C gene localised downstream of *tre*R, coding for the trehalose operon repressor (UIR13_07730). These elements are located in a putative mobile element, consistent with the presence of several transposases.

The variety of carbohydrate metabolism related genes in the *L. fermentum* CRL2085 genome is significant in terms of its potential versatile capability to gastrointestinal microhabitats and interactions with human hosts; hence, it may improve its survivability, competitiveness, and persistence. Moreover, in vitro carbon source utilisation by *L. fermentum* CRL2085 evaluated by the Biolog AN system showed that from the utilised 42 carbon sources, α‐ketovaleric acid, palatinose, and pyruvic acid methyl ester were the greatest metabolised. Alpha‐ketoacids are central intermediates that are derived from amino acids by the transaminase pathway and generate potent flavour compounds (Smit et al. 2005). Palatinose or isomaltulose, as a sucrose derivative, can be completely degraded to glucose and fructose by disaccharidase (Boonyanit et al. 2011). In addition, a high rate of pyruvic acid and pyruvic acid methyl ester was also consumed by *L. fermentum* CRL2085, which plays a key role for cellular biosynthesis.

The survival of orally administered probiotics is greatly compromised by the unique environment of the GIT, where they are exposed to a plethora of harsh physicochemical conditions (Bustos et al. 2024). In the *L. fermentum* CRL2085 genome, we identified an abundance of genes encoding proteins associated with its probiotic properties (Table S6). Mechanisms developed by probiotics in response to GIT stress conditions involved the modification of cell envelope and membrane lipids, alteration of metabolic pathways, and over‐expression of stress proteins for macromolecule repair (Bustos et al. 2024). Therefore, protein quality control, including refolding or degradation of damaged proteins, plays an indispensable role under stressed conditions; the synthesis of chaperones and proteases is quickly induced to cope with this situation. Molecular chaperones are a special class of heat shock proteins (Hsp), while the tasks of chaperones are to protect functional proteins and to refold misfolded ones; proteases provide the last line of defence by removing irreversibly damaged proteins, after which the amino acids are recycled (Papadimitriou et al. 2016). Among them, 11 genes were predicted for stress response, such as the system *dnaK/dnaJ*, a well‐conserved bacterial chaperone from the Hsp70 family that can efficiently refold misfolded proteins, while another predicted refolding system is the molecular chaperon and co‐chaperon *groEL/groES* coding genes (Hsp60 family; Edkins and Boshoff 2021). In agreement, the presence of these genes was also found in *L. fermentum* LAB‐1 (Hossain 2022) and other lactobacilli and bifidobacteria, as described by Mills et al. (2011). A putative thioredoxin *trxA* and thioredoxin‐disulfide reductase *trxB* genes were found in *L. fermentum* CRL2085, in agreement with that reported for *Lacticaseibacillus casei* and *Lpb. plantarum* (Serata et al. 2012; Serrano et al. 2007). In addition, the two genes *hslO* and *tpx* encoding proteins redox‐regulated chaperone and thiol‐specific peroxidase proteins were predicted in CRL2085. These genes emphasise the protection from oxidative stresses, playing a pivotal role in aerobic growth. In addition, four genes coding for Clp family proteins were predicted in the genome of *L. fermentum* CRL2085. Clp proteins are involved in the main system for general protein turnover in LAB and other low GC bacteria. The ATP‐dependent ClpP protease is a two‐component protease consisting of a *clpP*‐encoded serine peptidase subunit and a Clp ATPase subunit (Frees et al. 2007). Among them, genes coding for ATP‐dependent Clp protease ATP‐binding subunits ClpX, ClpE, ClpB, and ClpA/ClpB proteases were annotated in the CRL2085 strain. Some Clp ATPases can interact with ClpP, while others function as independent chaperones. As reported by Papadimitriou et al. (2016), the genes for *clpX*, *clpE*, and *clpB* are typically present in LAB. In coincidence, the genes encoding ClpX and ClpE proteins were reported in the genome of *L. fermentum* 222 (from cocoa bean fermentation) and ATC23271 (from human intestines) (dos Santos et al. 2021; Illeghems et al. 2015). In addition, a set of genes involved in acid and bile salts tolerance was predicted in the *L. fermentum* CRL2085 genome. Two encoding genes for glucose‐6‐phosphate isomerase, involved in acid and bile tolerance acting as an acid shock protein, were annotated in the *L. fermentum* CRL2085 genome, in agreement with what was detected in the *L. fermentum* ATCC23271 genome (dos Santos et al. 2021). A set of genes related to acid stress was also predicted; F0F1‐ATP synthases catalyse the most abundant physiological reaction in the cell; membrane‐bound enzymes use the energy derived from an electrochemical proton gradient for ATP formation. F0F1‐ATP synthase subunits encoding genes *atpD, atpA*, *atpF, atpE*, and *atpB*, as well as the *atpH* gene (ATP synthase F1 subunit delta) were predicted in the *L. fermentum* CRL2085 genome. ATP synthases are proteins mainly involved in acid tolerance, as they are associated with pH cytoplasmic regulation by ATP hydrolysis, which maintains pH homeostasis and protects cells from the damage induced by an acidic environment (Neupane et al. 2019). Concerning bile salts tolerance, *L. fermentum* CRL2085 showed a high number of genes (31); the prediction of two glucose‐6‐phosphate isomerase encoding genes *GPI* (converts glucose‐6‐phosphate to fructose‐6‐phosphate) was found, in agreement with that reported for *L. fermentum* ATCC23271 (dos Santos et al. 2021). In addition, seven putative genes coding for phosphotransferase system (PTS) were also identified in the CRL2085 strain. PTS is one of the major active sugar transport systems for bacteria. Several sugar‐specific PTS components were annotated, including mannose‐specific IIA, IIC, and IID components, sucrose‐specific IIB, cellobiose‐specific IIC, and trehalose‐specific IIA, as well as IIC components. The PTS system has the advantage of being energetically more efficient than other active transport systems since it is coupled with substrate‐level phosphorylation (Jeckelmann and Erni 2020). Similar PTS systems were present in *L. fermentum* ATCC23271 and LAB‐1 (Erni 2013; dos Santos et al. 2021; Hossain 2022). ABC transporter and proline/glycine betaine transporter permeases coded by *choS* and *gbuB* genes, respectively, were also predicted in CRL2085; they are involved in the acquisition of osmoprotectants such as choline, proline, glycine, and betaine to avoid osmotic stress in bacteria. The *arcD* gene, coding for arginine/ornithine antiporter, was also identified in *L. fermentum* CRL2085, in coincidence with that reported for *L. fermentum* ATCC23271, LAB‐1, and IMDO130101 (dos Santos et al. 2021; Hossain 2022; Verce et al. 2018); this amino acid membrane transporter of the electrochemical potential‐driven transport family is part of the arginine deiminase system (ADS) that facilitates arginine supply as ADS substrate, thereby contributing to environmental pH homeostasis and biologic fitness of bacteria. As a contribution to acid tolerance, a sodium proton antiporter transport protein encoding gene *yvgP* was present in the CRL2085 genome. Also, the genomic analysis predicted three genes, *ykgE*, *ykgF*, and *ykg*G, encoding for L‐lactate dehydrogenase (LDH) protein subunits, which are located within a gene cluster (*ykg*EFG); the LDH complex of proteins is encoded by the lactate permease operon, which may contribute to lactate catabolism and short chain fatty acids biosynthesis (Zhao et al. 2019).

Although a lower adhesion to Caco‐2 human intestinal cells was shown for *L. fermentum* CRL2085 compared to the *L. rhamnosus* GG strain, the in vivo outcomes previously reported by this strain (Mansilla et al. 2020, 2022, 2023, 2024) strongly suggest that it can successfully interact with the host environment. Indeed, three genes associated with adhesion were predicted in its genome (Table S6). The presence of *inlJ* and *mapA* genes, coding for the internalin J (MucBP domain) and the hydrolase collagen‐binding protein, was also reported in the genome of *L. fermentum* 3872 (Chatterjee et al. 2018). Since *L. fermentum* CRL2085 was demonstrated to produce EPS (Figure 3), it might directly adhere to the surfaces of intestinal surfaces promoting bacterial adhesion. In addition, two peptidoglycan‐binding LysM proteins related to the aggregation process were also predicted in coincidence with that reported for the *L. fermentum* ATCC23271 genome (dos Santos et al. 2021). Furthermore, we demonstrated that *L. fermentum* CRL2085 is able to produce EPS when grown in the presence of sucrose, a carbohydrate that can be present in the bovine diet and gut. EPS production may contribute to biofilm formation, which in turn could enhance mucosal adhesion in vivo.

In addition, EPS are known to enhance probiotic competence of LAB, protecting cell integrity in harsh conditions and providing resistance against antibiotics and phages, while also acting as prebiotic compounds. We identified several genes potentially involved in EPS biosynthesis. Detected genes included those encoding for sugar‐activating enzymes, one β‐phosphoglucomutase (*β‐pgm*), an UDP‐glucose‐4‐epimerase (*galE*), an UTP‐glucose‐1‐phosphate uridylyl‐transferase (*galU*), involved in the activation of precursor molecules, and *epsD* and *epsB* coding for two tyrosine‐kinase proteins as previously described for *L. fermentum* MC1 (breast milk), D2 (cow milk) and LAB‐1 (yogur‐like beverage) (Butorac et al. 2021; Čuljak et al. 2024; Hossain 2022). In addition, in the *L. fermentum* CRL2085 genome we identified a gene coding for a GH70 (UIR13_07035), a predicted dextransucrase of 1638 aa with a KxYKxGKxW signal peptide domain (pfam 19258), three putative cell wall binding repeats, and two choline binding repeats, putatively responsible for the EPS production detected when the strain was grown on MRS supplemented with sucrose. Furthermore, three putative EPS clusters were mapped in the *L. fermentum* CRL2085 genome. The first, located in contig_42, comprised two transposase genes (UIR13_04960 and UIR13_05055), and genes coding for a Wzz domain containing protein, which has chain length regulation role, capsule biosynthetic proteins, GTs, EpsG family protein, and polysaccharide biosynthesis C‐terminal domain‐containing protein (UIR13_05070). The second cluster, although mapped in several contigs, contained several GTs of families 2 and 4, and one galactofuranosyltransferase. Both these two clusters are similar to the EPS clusters described in *L. fermentum* L2, an EPS producing strain isolated from Argentine cheese (Harris et al. 2018). The third cluster is putatively involved in O‐antigen glycans biosynthesis and comprised genes coding for a sugar transferase (UIR13_00805), a Wzz domain containing protein (UIR13_00820), one GT2 family protein (UIR13_00830), *glf* coding for UDP‐galactopyranose mutase (UIR13_00840), and one flippase (UIR13_00840).

The probiotic strain *Lactobacillus johnsonii* FI9785, an E producer of exopolysaccharides (EPS) studied in poultry, has been reported to be effective as a competitive exclusion agent against *Campylobacter jejuni* . The structure and biosynthesis of dextran and Heptanose‐containing polysaccharides (HePS) in this strain, as well as the potential role of these polymers in biofilm formation and host colonisation as a protective mechanism against the hostile intestinal environment were described in previous studies (Dertli et al. 2013, 2015; Werning et al. 2022). The EPS‐producing strains *Ligilactobacillus salivarius* BIS312 and BIS722 were tested combined with their purified EPS, aiming at demonstrating the benefits of potential synbiotic applications in poultry feeding (Bikric et al. 2022). In addition, the in vitro antioxidant, antibacterial, and anti‐biofilm activities of the HePS polymer produced by the probiotic strain *Lactobacillus gasseri* F4, isolated from the gastrointestinal tract of free‐range chickens, have been demonstrated (Rani et al. 2017). Therefore, the ability of *L. fermentum* CRL2085 to produce EPS is a key factor in its probiotic potential. The use of EPS‐producing LAB as dietary supplements in animals intended for food production could help prevent or reduce the spread of pathogens during the primary production stage, thereby avoiding the use of antimicrobials in food production systems.

Biofilms or adherent structured microbial communities increase resistance to gastrointestinal environment‐related conditions. Eleven genes for biofilm production have been predicted in the genome of CRL2085; among them *comGC* and *comGB* coding for proteins Type IV pili, which are dynamic filaments on the surface of many bacteria with essential roles in attachment, colonisation, and biofilm formation. The genes *comEA*, *comEB*, and *comCC* encode proteins involved in DNA transport through the cell membrane in natural transformation/horizontal gene transfer, while *comFA* is the motor powering DNA transport during natural transformation (Burghard‐Schrod et al. 2022; Foster et al. 2022). In addition, we detected elements of the LuxS/AI‐2 quorum sensing system, which mediates interspecies and intraspecies signalling, regulates the probiotic activities of LAB, and is associated with biofilm formation, pathogenicity, and resistance to harsh conditions (Meng et al. 2022).

**FIGURE 5:**
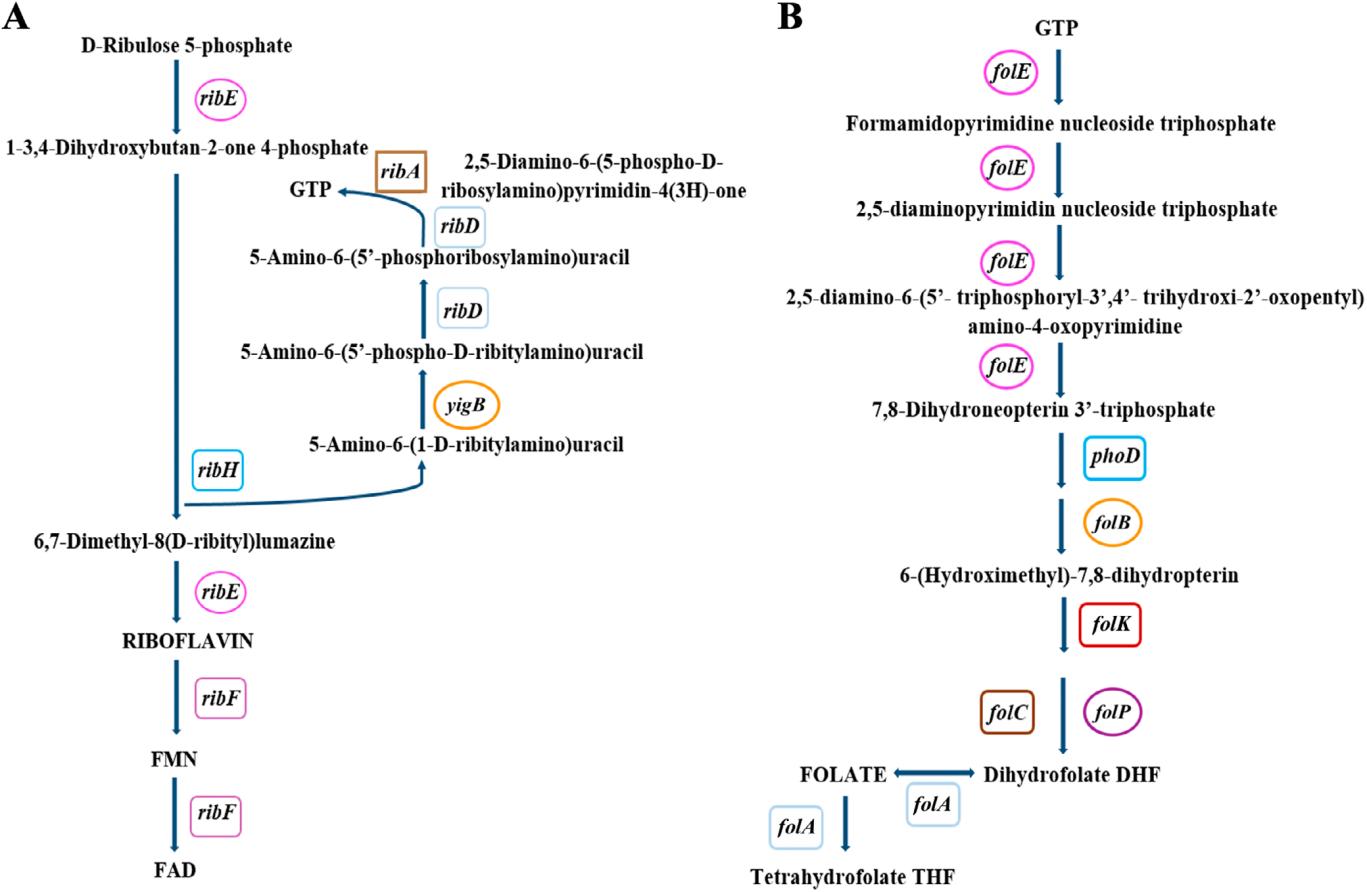
(A) Riboflavin and (B) folate biosynthetic pathway reconstructed in the genome of *L. fermentum* CRL2085.

In *L. fermentum* CRL2085 genome, genes associated with nutritional features, such as those for B‐group vitamins such as riboflavin (vitamin B2) and folate (vitamin B9) biosynthesis, as well as feruloyl‐esterase hydrolase, were predicted. Complete pathways for riboflavin (*ribA*, *ribB*, *ribD, ribE*, *ribF* and *yigB*) and folate (*folA*, *folB*, *folK*, *folP, folC, folE and phoD*) biosynthesis were reconstructed (Figure 5). Riboflavin biosynthesis has a convergent pathway with the initial substrates of the individual branches being guanosine triphosphate and d‐ribulose 5‐phosphate, both prevalent metabolites. In LAB, riboflavin synthase encoding genes were clustered on a *rib* operon, and its products RibA, RibB, RibD/G, and RibE/H catalyse the conversion of GTP and 5‐phosphate ribose into riboflavin (Levit et al. 2020) (Figure 5A). Additionally, *ribF* and *yigB* genes coding for bifunctional flavokinase able to dephosphorylate FMN (flavin‐mononucleotide), involved in oxidation–reduction reactions at all cellular levels (Averianova et al. 2020) were also predicted. Folate biosynthesis in lactobacilli requires eight enzymes involved in the conversion of the guanosine triphosphate (GTP) precursor into tetrahydrofolate (THF) polyglutamate (Mahara et al. 2023). Folate biosynthesis proceeds through three main building blocks, namely the pteridine moiety (6‐hydroxymethyl‐7,8‐dihydropterinpyrophosphate (DHPPP)), 4‐aminobenzoic acid (p‐aminobenzoic acid or PABA), and glutamate (Figure 5B). Since most LAB are unable to synthesise PABA and glutamate (Rossi et al. 2011), they must be supplied in the medium. Thus, the folate biosynthetic pathway can be split into two phases, that is, the formation of the pteridine moiety (DHPPP) and the combination of the three constituents of folate. Another interesting nutritional prediction in CRL2085 probiotic strain was the presence of two genes encoding feruloyl esterase (FAE) proteins, in coincidence with those previously described by Uezen et al. (2020). The predicted product was annotated as an aspartate‐semialdehyde dehydrogenase, which catalyses the NADPH‐dependent formation of L‐aspartate‐semialdehyde (L‐ASA) through the reductive dephosphorylation of L‐aspartyl‐4‐phosphate. The second gene predicted encoded esterases/lipases belonging to the α/β‐fold hydrolase family, exhibiting the classical serine nucleophilic motif (GxSxG) described for some carboxyl esterases. Feruloyl esterase is a hydrolase subclass acting on carboxylic esters. Indeed, the FAE enzyme acts on phenolic acids and polysaccharides involved in the cross‐linking of hemicellulose and lignin in plant cell walls, catalysing the formation of ferulate ester bonds, resulting in the release of various free hydroxycinnamic acids (HCA) or their dimers. In addition to its ability to degrade hemicellulose (silage), ferulic acid is a cinnamic acid derivative exhibiting strong antioxidant activity (Zheng et al. 2024). It has been observed that silage inoculants producing feruloyl esterase (FAE) have great potential to improve silage digestion efficiency, breaking the bonds between lignin and carbohydrates in the cell walls of forages, thus releasing ferulic acid from arabinoxylans during the ensiling process; indeed, the cell wall digestion degree in the rumen is strongly influenced by the amount of ferulic acid (Guo et al. 2022; Uezen et al. 2020).

The integrated genomic and phenotypic approach adopted in this study highlights the potential of *L. fermentum* CRL2085 as a probiotic candidate for cattle feed applications. However, further studies are necessary to evaluate its persistence in vivo models of the bovine gastrointestinal tract, in which the immunomodulatory effect of this strain was already proved (Mansilla et al. 2020). Although the strain exhibited only moderate adhesion to Caco‐2 cells, this limitation may not fully reflect its ability to interact with bovine intestinal cells. Furthermore, as with any probiotic strain, the optimisation of administration strategies, including dosage, delivery methods (e.g., encapsulation), and formulation, and the potential synergy with prebiotic or other probiotic components is essential. In addition, long‐term in vivo studies are required to comprehensively assess its effects on animal health, immune modulation, and gut microbiota composition, ultimately refining its application in livestock systems.

## 5 Conclusions

This study provides a comprehensive genomic and phenotypic characterisation of *Limosilactobacillus fermentum* CRL2085, a strain previously shown to improve the nutritional status and productivity parameters in feedlot cattle, highlighting several traits of functional and nutritional relevance that support its use as a probiotic in cattle feed. The presence of genes involved in acid and bile salt tolerance, oxidative and osmotic stress response, and adhesion supports the strain's capability to survive and function in the gastrointestinal tract of ruminants.

The ability of *L. fermentum* CRL2085 to metabolise a broad spectrum of plant‐derived carbohydrates, including those abundant in cattle feed (e.g., starch, cellobiose, maltodextrins), and to synthesise B‐group vitamins such as folate and riboflavin, supports its potential for improving nutrient availability, feed digestibility, and gut health in cattle. These features, along with its capacity to produce EPS with sucrose, which contribute to biofilm formation, may also enhance microbial stability and colonisation in the bovine gut environment.

The genomic and pangenomic analysis presented in this work will contribute to expanding the knowledge on *L. fermentum* species for its potential use in sustainable animal production systems. In addition, when combined with phenotypic validation, this combined approach provides a robust framework for the rational selection of probiotics for livestock applications.

The absence of horizontally acquired antimicrobial genes validated the safety assessment of the strains and positioned it as a promising candidate for inclusion in feed formulations as a natural alternative to antibiotic growth promoters, contributing to safer and more sustainable meat production systems. This aligns with current regulatory trends and growing consumer demand for antibiotic‐free livestock products.

Further studies will be focused on optimising delivery systems and synergistic formulations with prebiotics, to improve the viability and in vivo efficacy of the strain under feedlot conditions, as well as evaluating its long‐term effects on animal performance, immune modulation, and microbiome composition.

## Author Contributions

**Cecilia M. Aristimuño Ficoseco:** conceptualization, data curation, investigation, visualization, writing – original draft. **Daniele Chieffi:** formal analysis, investigation, visualization. **Marco Montemurro:** formal analysis, investigation, visualization. **Annarita Bavaro:** investigation. **Carlo G. Rizzello:** resources. **Maria E. F. Nader‐Macias:** visualization. **Silvina Fadda:** methodology, funding acquisition, resources, project administration. **Francesca Fanelli:** conceptualization, methodology, data curation, investigation, formal analysis, supervision, visualization, writing – original draft, writing – review and editing. **Vincenzina Fusco:** methodology, funding acquisition, project administration, resources. **Graciela M. Vignolo:** conceptualization, writing – original draft, writing – review and editing.

## Conflicts of Interest

The authors declare no conflicts of interest.

## Supporting information


**Figure S1:** Genome‐based phylogeny of *L. fermentum* CRL2085. The tree was inferred by using the maximum likelihood method RAxML with progressive refinements. *Lactobacillus oligofermentans* DSM 15707 was used as an outgroup. The tree is drawn to scale. Support values are represented by scaled circles at each node.

**Figure S2:** Accessory‐based tree of *L. fermentum* strains. Distance tree was generated by hierarchical clustering from presence/absence binary matrix of accessory genes. The dendrogram on the left represents hierarchical clustering based on Euclidean distance. Isolation source and geographical origin are indicated by a square box close to the strain name, according to the legend.

**Table S1:** List of strains and genomes used in this study.

**Table S2:** Genomic Island identified in the genome of *L. fermentum* CRL2085.

**Table S3:** Transposases identified in the genome of *L. fermentum* CRL2085.

**Table S4:** CRISPR‐Cas elements identified in the genome of *L. fermentum* CRL2085.

**Table S5:** Matrix of the average nucleotide Identity among *L. fermentum* strains.

**Table S6:** Genes associated with probiotic function identified in the genome of *Limosilactobacillus fermentum* CRL2085.

## Data Availability

The data that support the findings of this study are available from the corresponding author upon reasonable request.
