# Supplementary figures and images for: Genomic Characterisation of Limosilactobacillus fermentum CRL2085 Unveiling Probiotic Traits for Application in Cattle Feed
Source: Environ Microbiol Rep. 2025 Sep 8;17(5):e70176. doi: 10.1111/1758-2229.70176 (PMC12415445; doi:10.1111/1758-2229.70176)

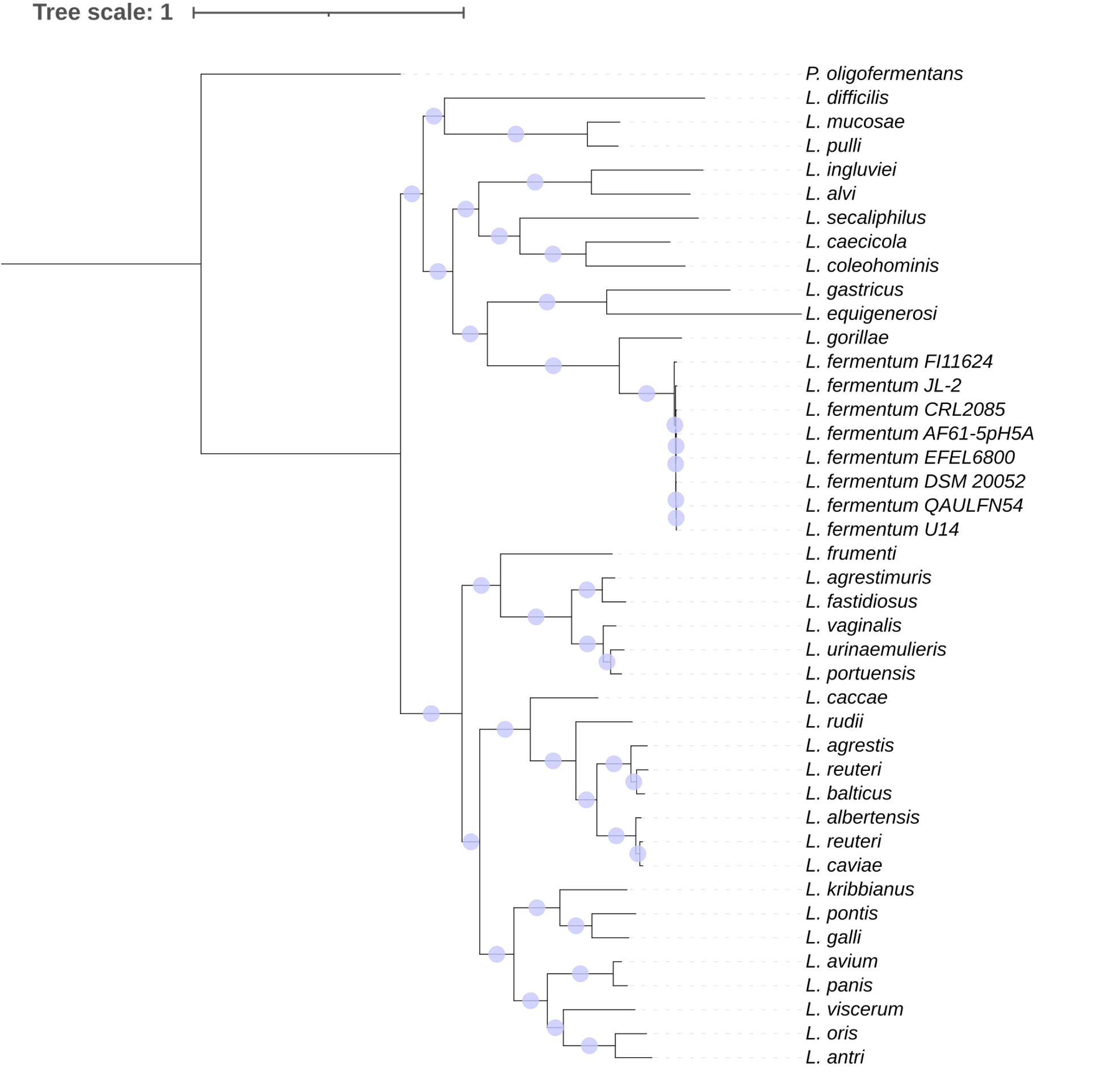

Supplement: Supplementary file 1 — Figure S1: Genome‐based phylogeny of L. fermentum CRL2085. The tree was inferred by using the maximum likelihood method RAxML with progressive refinements. Lactobacillus oligofermentans DSM 15707 was used as an outgroup. The tree is drawn to scale. Support values are represented by scaled circles at each node. [file EMI4-17-e70176-s006.png]

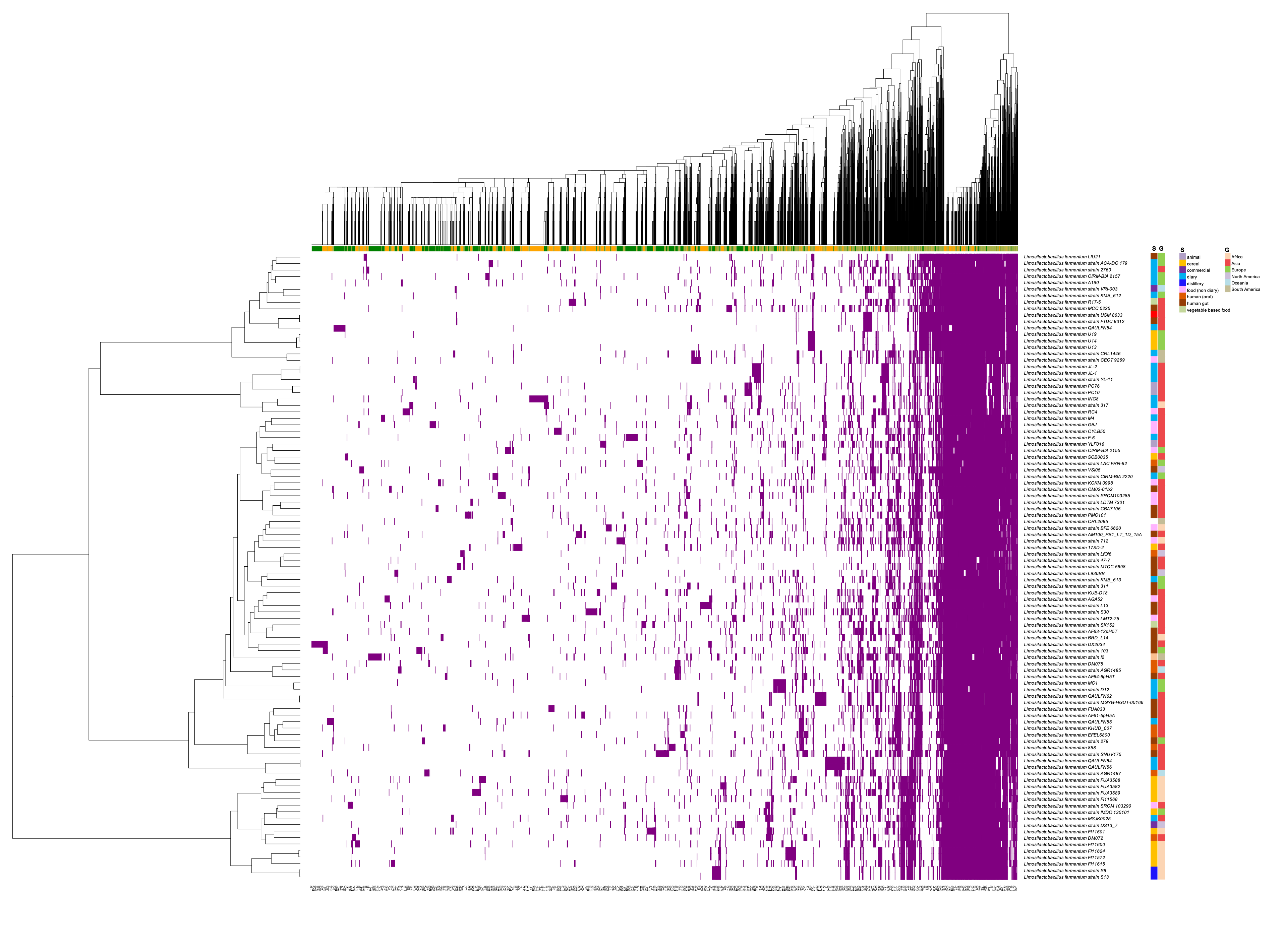

Supplement: Supplementary file 2 — Figure S2: Accessory‐based tree of L. fermentum strains. Distance tree was generated by hierarchical clustering from presence/absence binary matrix of accessory genes. The dendrogram on the left represents hierarchical clustering based on Euclidean distance. Isolation source and geographical origin are indicated by a square box close to the strain name, according to the legend. [file EMI4-17-e70176-s005.png]
